# Channel-specific input/output transformations arising from the interaction between dynamic synapses and subthreshold oscillations

**DOI:** 10.1186/1471-2202-16-S1-P274

**Published:** 2015-12-18

**Authors:** Roberto Latorre, Joaquin J Torres, Pablo Varona

**Affiliations:** 1 Dpto. de Ingeniería Informática, Escuela Politécnica Superior, Universidad Autónoma de Madrid, 28049 Madrid, Spain; 2 Dpto. de Electromagnetismo y Física de la Materia, and Institute Carlos I for Theoretical and Computational Physics, University of Granada, Granada, Spain

## 

Subthreshold oscillations are observed in a wide variety of neurons in the nervous system (e.g. see [1,2]). They typically appear associated to resonance phenomena that allow, for instance, the implementation of intrinsic memory mechanisms for the detection of specific spike sequences in single neurons and neural networks [3-5]. On the other hand, synaptic transmission and, therefore, the corresponding input/output transformation in the postsynaptic cell are affected by recent presynaptic activity. Dynamic synapses modulate incoming information increasing (facilitation) or decreasing (depression) the postsynaptic response. This modulation provides additional characteristic time scales to the single neuron and network processing [6,7]. In the context of a neuron displaying subthreshold oscillations, the interplay between these additional time scales and the characteristic intrinsic fast and slow dynamics underlying the neuron's oscillatory activity can significantly affect its input/output transformation.

Neuronal intrinsic subthreshold oscillations and dynamic synapses underlie several computational properties both at the single neuron and the network levels. Traditionally, intrinsic oscillations and dynamic synapses have been studied separately and their interaction has attracted almost no attention. In this work, we use a conductance-based neuron model and a dynamic synapse to investigate how intrinsic subthreshold oscillations and the short-term plasticity of a depressing synapse act together to shape its resonant properties and the corresponding input/output transformation. Our results suggest that factors such as the maximum hyperpolarization level, the oscillation amplitude and frequency or the resulting firing threshold can be modulated by synaptic depression. This shapes the postsynaptic neuron's resonant properties arising from the subthreshold oscillation and leads to complex channel-specific input/output relations. Thus, a low-cost modification in synaptic parameters can produce a significant different response. This complex synaptic-dependent input/output transformation allows the implementation of cost-effective information discrimination mechanisms in single neurons by just tuning the depression level of the synaptic channel without modifying the intrinsic neural dynamics.
